# The long-term impact of Spain's 2010 Anti-Smoking Law: A counterfactual and prospective time-series analysis

**DOI:** 10.3934/publichealth.2026011

**Published:** 2026-01-29

**Authors:** Juan Manuel Martín-Álvarez, Aida Galiano, Brenda Vázquez-La Hoz, Serhiy Lyalkov

**Affiliations:** Departments of Quantitative Methods for Economics and Business and Marketing and Market Research. Faculty of Economics and Business. Universidad Internacional de La Rioja, Logroño, Spain

**Keywords:** Anti-Smoking Law, tobacco control, counterfactual analysis, time-series forecasting, machine learning, public health policy, structural change, Spain

## Abstract

This study evaluated the impact of Spain's 2010 Anti-Smoking Law on cigarette sales using a hybrid counterfactual and forecasting framework that combines econometric and machine learning models. Monthly provincial data observed from January 2005 to August 2025 are used to estimate the historical effects of the law (2011–2013) and to generate prospective projections under alternative scenarios for the period 2025–2027. Six time-series models—ETS, Harmonic, NNAR, SARIMA, STL_AR, and STLM—were compared using a multi-metric validation scheme to ensure robustness. Results indicate that the 2010 law generated an immediate and persistent reduction in cigarette sales throughout Spain. Across models, the cumulative national decline over 2011–2013 ranged between 0.37 and 2.45 billion cigarette packs, equivalent to a 15%–30% contraction in the tobacco market. Projections from September 2025 to December 2027 suggest continued stabilization or slight decreases in cigarette sales, indicating the long-term persistence of the law's impact. Methodologically, the study demonstrates that hybrid time-series and machine learning ensembles outperform single-model approaches in capturing structural breaks, nonlinearities, and seasonality shifts associated with behavioral regulation. The results confirm the effectiveness of comprehensive tobacco control policies in achieving sustained public health gains. Overall, Spain's experience exemplifies how evidence-based regulation can produce lasting declines in harmful consumption and provides a replicable framework for evaluating future public health interventions.

## 1. Introduction

Tobacco use remains one of the leading preventable causes of disease and death worldwide. According to the World Health Organization (WHO), tobacco kills more than eight million people each year, including over one million non-smokers exposed to secondhand smoke. Beyond its direct health consequences, tobacco consumption deepens social inequalities, increases healthcare expenditure, and burdens national productivity. Public health policies aimed at reducing tobacco use—through taxation, advertising bans, and smoke-free environments—are among the most cost-effective strategies to mitigate this global burden.

### 1.1. Tobacco control and public health context: legislative impact and marketing adaptation

The evolution of tobacco control has been strongly guided by the WHO Framework Convention on Tobacco Control (FCTC) and the MPOWER package, which emphasize evidence-based regulation of advertising, product sales, and public exposure [1]–[3]. Spain's adoption of Law 28/2005 (partial restrictions) and Law 42/2010 (comprehensive bans) situates it within this international framework, marking a transition from moderate regulation to a public health protection model. These legislative milestones have provided a quasi-natural experiment for examining the real-world impact of policy interventions on tobacco markets and public behaviors [4],[5].

Empirical studies consistently demonstrate that legislative restrictions on tobacco advertising and consumption yield measurable declines in sales and smoking prevalence. In Spain, Pinilla et al. [6] documented an 11% immediate reduction in cigarette sales following the implementation of the 2011 comprehensive ban, while Galiano et al. [7] and Del Arco-Osuna et al. [5] observed convergence across provinces, with decreasing disparities in consumption patterns after regulatory harmonization. Complementary findings by Almeida et al. [8] suggest that these policies not only reshaped the cigarette market but also redefined competitive dynamics, forcing tobacco companies to reorient marketing strategies away from brand innovation and toward behavioral mimicry and social diffusion.

At the global level, Fu et al. [9] and Peruga et al. [3] provided robust evidence that comprehensive anti-tobacco measures contribute to both public health gains and macroeconomic benefits, demonstrating a dual dividend: reduced morbidity and modest GDP growth through enhanced labor productivity. These outcomes highlight how health regulation can yield cross-sectoral advantages, positioning tobacco control as a public investment rather than merely a restrictive measure. Akter et al. [1] and DeCicca et al. [4] further argued that the economic evaluation of anti-smoking laws should integrate behavioral models to capture adaptive consumer responses, providing a theoretical basis for predictive modeling of policy impacts.

Despite the formal prohibition of tobacco advertising, marketing persists through indirect or symbolic channels. Feliu et al. [10] found that tobacco imagery remains frequent in Spanish cinema, often portraying smoking as socially normative or aesthetically desirable. This indirect exposure sustains brand salience and undermines the denormalization efforts central to tobacco control. The persistence of such "dark marketing” echoes findings from other countries, where the tobacco industry adapts through packaging design, sponsorship surrogates, and cross-media visibility. Rogers et al. [11] identified similar tactics in the United States: despite local bans on flavored and menthol products, companies introduced “concept-named” alternatives, exploiting regulatory loopholes and consumer ambiguity. These adaptations illustrate the co-evolutionary tension between regulation and marketing, a dynamic that must be accounted for in predictive models of public health policy effectiveness.

Public health research underscores that the success of anti-tobacco legislation depends not only on legal prohibitions but also on institutional implementation and cultural diffusion. Wang et al. [12] showed that smoke-free campus policies in the United States doubled between 2012 and 2017, with over 2000 colleges adopting tobacco-free regulations. These interventions not only reduced secondhand smoke exposure but also shaped youth norms, reinforcing non-smoking as a social default. Earlier evidence by Rigotti et al. [13] demonstrated that university smoking restrictions directly reduced cigarette consumption among students and increased cessation intentions, highlighting the importance of environmental reinforcement in policy outcomes. Similarly, Prayitno and Miekhel [14] argued that emerging-market regulations, while often fragmented, can achieve meaningful behavioral change when supported by education and consistent enforcement. Taken together, these studies affirm that policy design, enforcement capacity, and cultural context are as critical as the legal content itself. From a public health standpoint, understanding how individuals and industries adapt to regulation is essential for predicting long-term outcomes and avoiding unintended consequences such as illicit trade, product substitution, or symbolic advertising.

### 1.2. The need for predictive and counterfactual approaches in policy evaluation

Traditional policy evaluations in public health often rely on econometric or interrupted-time-series designs such as SARIMA or difference-in-differences. While these approaches capture average treatment effects, they are limited in handling nonlinearities, multi-level dependencies, and temporal drift—features intrinsic to consumer behavior and marketing adaptation. Machine learning provides a powerful complement by uncovering latent patterns in time-series data and generating counterfactual predictions under alternative policy scenarios. Leveraging machine learning for predictive risk assessment allows researchers to move beyond retrospective analysis toward prospective policy simulation. By constructing counterfactual series—what provincial sales would have been in the absence of the ban—these models enable quantification of both immediate and sustained policy impacts. Furthermore, they provide a framework to evaluate the risk of policy relaxation, such as how cigarette consumption might rebound if advertising restrictions were loosened or enforcement weakened.

Given that this study includes authors from marketing, it emphasizes the dual interface of public health regulation and market behavior. The tobacco market exemplifies how commercial communication influences health risks. By analyzing cigarette sales as behavioral signals shaped by policy, enforcement, and advertising restrictions, this research contributes to the literature on tobacco control and applied time-series policy evaluation. For public health, it provides evidence-based insights for evaluating the efficacy of legislation in reducing exposure and consumption. For marketing and communication, it reveals how regulatory contexts reshape consumer decision architectures and brand signaling under constraint. This interdisciplinary framing aligns with the broader call for translational public health analytics—the use of computational tools to anticipate, measure, and communicate the population-level impacts of behavioral and regulatory interventions.

Seasonal autoregressive integrated moving average (SARIMA) models have long been recognized as reliable approaches for forecasting epidemiological and socioeconomic time series. Their ability to capture both trend and seasonal fluctuations makes them suitable for modeling behavioral and policy-related phenomena in public health. As shown by Perone [15] and Dey et al. [16], SARIMA consistently outperforms traditional Autoregressive Integrated Moving Average (ARIMA) models in predicting medium-term public health dynamics by accurately reflecting cyclical and structural variations. The methodological rigor of SARIMA lies in its capacity to decompose complex temporal behaviors into autoregressive and seasonal components, allowing for transparent interpretation and reproducible forecasting. These properties are crucial for policy evaluation, as public health interventions such as anti-smoking legislation often produce delayed and cyclical effects in consumption and behavioral outcomes.

A critical evolution in public health analytics is the transition from forecasting to counterfactual inference—that is, estimating what would have happened in the absence of an intervention. This approach aligns with predictive risk assessment, providing decision-makers with evidence on both observed and hypothetical outcomes. Chenran et al. [17] introduced a counterfactual time-series framework comparing SARIMA, Long Short-Term Memory (LSTM), and Extreme Gradient Boosting (XGBoost) models, showing that SARIMA can serve as a robust baseline for causal inference when the intervention timing is well defined. In public health research, counterfactual modeling allows quantification of the policy effect in intuitive terms: differences between predicted (no-law) and observed (post-law) trajectories represent the magnitude of change attributable to the intervention. This structure directly parallels interrupted time-series and difference-in-differences frameworks but extends them through more flexible forecasting and seasonality handling.

The usefulness of SARIMA and related models for policy evaluation has been validated in the social sciences. Andueza et al. [18] and Galiano et al. [19] applied SARIMA-based counterfactual models to tourism and economic datasets to assess the effects of external shocks, such as the COVID-19 pandemic, showing that hybrid approaches yield robust counterfactual trajectories and improve accuracy relative to traditional econometric models. Similarly, Aquino et al. [20] demonstrated the applicability of SARIMA models to the evaluation of lockdown measures in Latin America, quantifying how behavioral restrictions influenced healthcare utilization rates. These applications collectively illustrate the growing acceptance of time-series counterfactuals as a methodological standard in impact evaluation.

While SARIMA offers interpretability and parsimony, recent advances in machine learning expand its predictive scope. Recurrent neural networks (RNNs), particularly LSTM architectures, and gradient boosting techniques such as XGBoost can capture nonlinear dependencies and structural breaks that exceed the representational capacity of linear models. Chenran et al. [17] found that LSTM models provided superior long-horizon predictions, whereas SARIMA performed best in short- to medium-term intervals, suggesting a complementary relationship between statistical and ML-based methods. The hybrid SARIMA–ML paradigm thus becomes a promising avenue for public health predictive modeling, leveraging the strengths of both interpretability and flexibility in settings where behavioral data—such as tobacco sales—exhibit structured seasonality and stochastic volatility.

In this study, the use of SARIMA and machine learning techniques enables the construction of counterfactual trajectories of cigarette sales at the provincial level in Spain. By comparing these predicted (no-ban) scenarios against observed data, it becomes possible to isolate the causal effect of anti-tobacco laws on consumption behavior. This methodology not only measures historical effectiveness but also extends to policy simulation—predicting the outcomes of potential future interventions, such as packaging standardization or digital advertising restrictions. The capacity to simulate hypothetical futures makes these models invaluable for strategic public health risk assessment, offering policymakers the ability to anticipate the magnitude and distribution of potential policy effects before implementation.

From an interdisciplinary perspective, this modeling framework connects three complementary domains: public health, through the measurement of intervention outcomes; econometrics and data science, through the development of counterfactual predictive tools; and marketing, by modeling the behavioral consequences of advertising bans and consumption shifts. As demonstrated by Galiano et al. [21], adopting counterfactual and machine learning techniques enhances the capacity to interpret both statistical impacts and behavioral mechanisms underlying consumer adaptation. In the tobacco market, this means assessing how advertising prohibitions and price changes interact with consumer inertia, substitution patterns, and regional heterogeneity—all crucial for effective health communication and equitable policy design.

Ultimately, the integration of SARIMA and machine learning models in counterfactual frameworks represents a paradigm shift in public health analytics. Instead of relying solely on retrospective evidence, this approach enables anticipatory governance—quantifying risks, projecting alternative outcomes, and identifying vulnerable populations before harm occurs. By leveraging high-frequency data and computational modeling, public health moves toward a predictive discipline, where the effectiveness of interventions like smoking bans, taxation, or advertising restrictions can be simulated, validated, and optimized in near-real time.

### 1.3. Beyond SARIMA: alternative machine learning and structural models

Although SARIMA and other linear time-series models have proven valuable, their assumptions often limit their ability to capture complex nonlinear behaviors in public health data. Recent advances in computational modeling have opened the door to more flexible predictive paradigms combining interpretability and adaptability. Li et al. [22] emphasized that nonlinear models such as LSTM and ensemble approaches outperform classical models in scenarios where external shocks or behavioral adaptations modify the time-series structure. In their study on post-pandemic recovery dynamics, hybrid LSTM–ARIMA models achieved lower forecasting error than traditional SARIMA, especially when policy interventions altered behavioral patterns.

Toharudin et al. [23] provided an illustrative example of how machine learning can be used to evaluate health interventions. Using extreme learning machines (ELM), multilayer perceptrons (MLP), and auto-regressive neural networks (NNAR), they forecasted COVID-19 trajectories during and after social restrictions in East Java, with MLP yielding the best short-term accuracy. Beyond predictive performance, their models captured nonlinear policy effects, offering implicit counterfactual assessments. Similarly, Toharudin et al. [24] combined neural networks and Bayesian modeling to quantify the impact of local interventions and vaccination rollout in Jakarta, showing that hybrid ML approaches can approximate causal inference when data constraints make traditional econometrics unreliable.

In parallel, Butkevych et al. [25] proposed an advanced Bayesian structural time series (BSTS) framework for counterfactual prediction in policy evaluation. This method integrates Bayesian inference with dynamic regression, allowing estimation of latent trends, structural breaks, and intervention effects under uncertainty. Compared with deterministic models like SARIMA, BSTS offers probabilistic counterfactuals and quantifies uncertainty, making it ideal for evaluating tobacco legislation where economic and behavioral mechanisms interact dynamically across time and space.

Beyond prediction, interpretability has become central to modern counterfactual models. Gunter [26] argued that explainable machine learning—using SHAP or partial dependence plots—bridges the gap between black-box predictions and policy relevance by identifying which variables most influence predicted outcomes. This interpretability transforms machine learning from a forecasting exercise into a diagnostic tool that helps policymakers understand when and why interventions succeed or fail.

The synthesis of these studies suggests that methodological pluralism—combining SARIMA, ML, and Bayesian models—provides the most robust framework for causal prediction in public health. SARIMA ensures temporal coherence and interpretability; neural and ensemble models capture nonlinear adaptation; and Bayesian structural approaches quantify uncertainty and enable scenario-based simulation. Together, these approaches allow researchers to construct multi-model counterfactual systems that simulate both observed and hypothetical realities. In the context of tobacco control, such systems can forecast the differential effects of policy variations, including taxation, marketing restrictions, or packaging regulations, under different enforcement or socioeconomic conditions.

The integration of these advanced models signals a conceptual transition from post-hoc evaluation to predictive policy analytics. By combining high-frequency data with hybrid ML and Bayesian models, researchers can simulate future risk trajectories of tobacco consumption under various regulatory regimes. This shift toward prediction enables anticipatory governance, aligning with the editorial theme of Leveraging Machine Learning for Predictive Public Health Risk Assessment. The present study situates itself at this intersection, using time-series data, hybrid models, and counterfactual inference to estimate and predict the impact of Spanish anti-tobacco legislation—bridging epidemiological evaluation, marketing analytics, and computational public health.

This study is guided by three closely related research questions. First, what is the magnitude and persistence of the impact of Spain's comprehensive smoking ban (Law 42/2010) on legal cigarette sales at the provincial and national levels? Second, to what extent are the estimated effects robust across alternative counterfactual specifications, including both classical time-series models and machine-learning approaches? Third, how are cigarette sales expected to evolve in the medium term under a continued policy regime, according to prospective model-based projections?

These questions motivate the empirical strategy adopted in the paper. The first question is addressed using an interrupted time-series counterfactual framework applied to high-frequency provincial data. The second is examined through a systematic comparison of results across multiple econometric and machine-learning models. The third is explored using prospective scenario-based forecasting, allowing us to assess the medium- to long-term implications of the observed structural break.

In addition, this study contributes to the empirical literature on tobacco control policies in several important ways. While most existing evaluations rely on annual or highly aggregated data, we exploit high-frequency monthly provincial data covering nearly two decades, which allows us to capture both immediate and persistent responses to policy interventions. Moreover, we adopt a hybrid counterfactual framework that combines classical time-series models with machine-learning approaches, enabling a systematic comparison of alternative specifications and reducing reliance on any single modeling paradigm.

Beyond retrospective impact evaluation, the paper also incorporates a prospective dimension, using validated forecasting models to project cigarette sales under alternative scenarios. This forward-looking component provides novel evidence on the persistence of policy-associated effects, a dimension that has received limited attention in the existing literature on smoking bans. Finally, by emphasizing robustness across models, metrics, and levels of aggregation, the analysis offers a comprehensive and transparent assessment of the effects of Spain's comprehensive smoking ban on legal cigarette sales.

Taken together, these contributions position the study at the intersection of policy evaluation, time-series econometrics, and applied machine learning, extending the existing evidence base on the long-term effects of tobacco control measures.

## 2. Materials and methods

### 2.1. Data

The data utilized in this study have been obtained from the Trade of Tobacco Commission of Spain. This dataset provides a monthly overview of legal cigarette sales across the 48 Spanish provinces from January 2005 to August 2025, resulting in a total of 11,904 observations.

To evaluate the historical and potential future effects of regulatory interventions, the time series has been divided into two empirical periods and one prospective simulation period, following the major legislative milestones in Spain's tobacco control policy: Act 28/2005 (implemented in December 2005) and Act 42/2010 (enforced in January 2011). The first period covers January 2005 to December 2010, encompassing 3456 observations (48 provinces over 72 months) and representing the phase of partial restrictions and early enforcement of Law 28/2005. The second period extends from January 2011 to August 2025, comprising 8448 observations (48 provinces over 176 months), which correspond to the era of comprehensive restrictions under Law 42/2010 and subsequent consolidations in public health policy.

In addition, a prospective counterfactual projection is developed from September 2025 onward to simulate the potential effects of a new anti-tobacco law currently under governmental consideration. This forecast scenario is generated using machine learning and SARIMA-based hybrid models trained on the historical data (2005–2025).

The resulting synthetic series estimates cigarette sales trajectories under two alternative conditions: Status quo (continuation of the existing regulatory framework beyond 2025) or new policy scenario (introduction of additional restrictions, e.g., plain packaging, extended smoke-free zones, or advertising bans in digital environments).

This forward-looking component extends the analytical scope of the study from retrospective evaluation to predictive public health risk assessment, allowing the estimation of the potential magnitude and direction of future policy impacts on tobacco sales and consumer behavior across Spanish provinces.

For analytical clarity, the time series is explicitly divided into three periods: (i) an observed period spanning January 2005 to August 2025; (ii) a historical counterfactual evaluation period (2011–2013), used to quantify the causal impact of Law 42/2010; and (iii) a projection period from September 2025 to December 2027, corresponding to prospective, model-based simulations.

### 2.2. Methodological framework

This study analyzes the evolution of cigarette sales in Spain and evaluates the causal and predictive impact of anti-tobacco legislation through hybrid time-series and machine learning models. The empirical framework builds on the official dataset of monthly cigarette sales provided by the Trade of Tobacco Commission of Spain, covering 48 provinces from January 2005 to August 2025.

The analysis distinguishes between two simulated scenarios. The status quo scenario represents a counterfactual trajectory in which cigarette sales evolve according to pre-intervention dynamics, assuming that the comprehensive smoking ban was not implemented and that pre-2011 trends and seasonal patterns continue unchanged. The new scenario incorporates the estimated post-2011 structural break and subsequent dynamics associated with Law 42/2010. All other aspects of the modeling framework are held constant across scenarios, ensuring that differences between trajectories reflect the introduction of the policy-related structural break rather than changes in model specification or assumptions.

All computations were conducted in R language [27] using a fully reproducible pipeline that automated data import, transformation, modeling, and forecasting. The workflow consisted of four main stages: (i) data structuring and preprocessing, (ii) model estimation and counterfactual construction, (iii) performance validation, and (iv) scenario simulation beyond 2025.

To enhance the transparency of the machine-learning component, we conduct a post-hoc interpretability analysis of the neural network autoregressive (NNAR) model. Unlike feature-rich machine-learning applications, the NNAR specification relies exclusively on lagged values and seasonal components of the outcome variable. Consequently, interpretability in this context focuses on the relative contribution of temporal lags and seasonal structures rather than on contemporaneous covariates.

Following recent applications of post-hoc interpretability in time-series and environmental forecasting models [28]–[32], we assess the sensitivity of NNAR predictions to perturbations in lagged inputs. The analysis shows that the model places dominant weight on recent lags and stable seasonal components, with diminishing influence from longer lag structures. This pattern is consistent with the behavior of classical autoregressive models and supports the internal coherence of the neural network forecasts.

This interpretability exercise is intended as a transparency and diagnostic tool rather than a causal decomposition. The causal interpretation of the policy effect remains grounded in the interrupted time-series and counterfactual framework, while the NNAR model contributes to robustness and forecasting accuracy.

#### 2.2.1. Model specification

To evaluate the temporal dynamics and counterfactual trajectories of cigarette sales, five distinct time-series models were estimated separately for each province. These include seasonal autoregressive integrated moving average (SARIMA), exponential smoothing state space model (ETS), harmonic regression, and neural network autoregression (NNAR).

These models were chosen for their complementary ability to capture linear trends, seasonality, nonlinearity, and structural uncertainty—key features in behavioral and policy-driven time series. The methodological foundation follows standard forecasting literature [33]–[35].

SARIMA models (Eq 1) account for both seasonal and non-seasonal dependencies [33]:

$$\Phi_{P}(B^{s})\varphi_{p}(B){\left(1-B\right)}^{d}{\left(1-B^{s}\right)}^{D}y_{t}=\Theta_{Q}(B^{s})\theta_{q}(B)\varepsilon_{t}$$ (1)

where *B* is the backshift operator, and (p, d, q), (P, D, Q), and s refer to the non-seasonal and seasonal orders and frequency.

ETS models (Eq 2) decompose the series into error, trend, and seasonal components using exponential smoothing [34]:

$$y_{t}=\left(l_{t-1}+b_{t-1}\right)s_{t-m}+\varepsilon_{t}$$ (2)

with *l_t_*, *b_t_*, and *s_t_* denoting the level, trend, and seasonality terms.

Harmonic regression models (Eq 3) seasonality using Fourier terms [34]:

$$y_{t}=\beta_{0}+\beta_{1}t+\text{Σ}\left[\alpha_{k}sin\left(2\pi kt/s\right)+\gamma_{k}cos\left(2\pi kt/s\right)\right]+\varepsilon_{t}$$ (3)

NNAR models (Eq 4) are feed-forward neural networks trained on lagged observations [35]:

$$y_{t}=f\left(y_{t-1},y_{t-2},\ldots ,y_{t-p}\right)+\varepsilon_{t}$$ (4)

where *f* is a nonlinear function trained via backpropagation.

STLM models (Eq 5) apply seasonal-trend decomposition using LOESS followed by ARIMA on the remainder [34]:

$$y_{t}=T_{t}+S_{t}+R_{t},where\;R_{t}∼ARIMA(p,d,q)$$ (5)

In all specifications, *y_t_* denotes observed cigarette sales at time *t*, where *t* = 1,...,*T* indexes monthly observations. The error term *ε_t_* captures random disturbances.

In exponential smoothing and state-space representations, *l_t_* denotes the level component, *b_t_* denotes the slope (trend) component, and *s_t−m_* denotes the seasonal component with seasonal period *m*.

In harmonic regression models, *t* denotes the time index, *s* represents the seasonal period (with *s* = 12 for monthly data), and *k* = 1,...,*K* indexes the order of the Fourier terms used to approximate seasonal patterns. The coefficients *α_k_* and *γ_k_* measure the contribution of the sine and cosine terms at each harmonic frequency.

In NNAR models, *p* denotes the number of lagged observations used as inputs, and *f*() represents a nonlinear function approximated by a feed-forward neural network trained via backpropagation.

In STL-based models, the observed series is decomposed as *y_t_* = *T_t_* + *S_t_* + *R_t_*, where *T_t_* denotes the trend component, *S_t_* the seasonal component, and *R_t_* the remainder. In the STL-AR specification, the remainder component follows an autoregressive process, while in the STLM specification it is modeled using an ARIMA (*p*, *d*, *q*) process.

For clarity and reproducibility, Table 1 summarizes the six time-series models estimated in the analysis, including a brief description of their structure and the main hyperparameters or selection procedures. Table 2 reports the evaluation metrics used to assess model performance, together with their definitions.

**Table 1.** Summary of estimated models.

Model	Type	Description	Key hyperparameters/selection
ETS	Statistical	Exponential smoothing state-space model	Automatic selection via AIC
Harmonic	Statistical	Regression with Fourier terms	Number of harmonics selected by AIC
NNAR	Machine learning	Neural network autoregressive model	Lag order and hidden units selected by validation
SARIMA	Statistical	Seasonal ARIMA model	Orders selected via AIC
STL_AR	Hybrid	STL decomposition + AR on remainder	AR order selected via AIC
STLM	Hybrid	STL decomposition + ETS	ETS selected via AIC

**Table 2.** Forecast evaluation metrics.

Metric	Formula	Interpretation
Mean absolute error (MAE)	$MAE=\left(\frac{1}{n}\right)\sum^{\text{​}}\left|y_{t}-\hat{y_{t}}\right|$	Measures the average magnitude of forecast errors in absolute terms. Lower values indicate higher accuracy.
Root mean squared error (RMSE)	$\text{RMSE}=\sqrt{\left(\frac{1}{n}\right)\sum^{\text{​}}{\left(y_{t}-\hat{y_{t}}\right)}^{2}}$	Penalizes larger errors more heavily than MAE, making it sensitive to large deviations.
Mean absolute percentage error (MAPE)	$MAPE=\left(\frac{100}{n}\right)\sum^{\text{​}}\left|\frac{y_{t}-\hat{y_{t}}}{y_{t}}\right|$	Average absolute percentage error; scale-free and comparable across series. Lower values indicate higher accuracy.
Symmetric mean absolute percentage error (sMAPE)	$SMAPE=\left(\frac{100}{n}\right)\sum^{\text{​}}\frac{2\left|y_{t}-\hat{y_{t}}\right|}{\left(\left|y_{t}\right|+\left|\hat{y_{t}}\right|\right)}$	Symmetric percentage-based error that reduces sensitivity to very small denominators in MAPE.
Mean absolute scaled error (MASE)	$MASE=\frac{MAE}{MAE_{naive}}$	Scales forecast accuracy relative to a naïve benchmark; values below 1 indicate improvement over the naïve model.
Median absolute percentage error (MdAPE)	$MdMAPE=100\left|\frac{y_{t}-\hat{y_{t}}}{y_{t}}\right|$	Median absolute percentage error; robust to outliers. Lower values indicate higher accuracy.
Bias	$Bias=\left(\frac{1}{n}\right)\sum^{\text{​}}(\hat{y_{t}}-y_{t})$	Captures systematic overestimation (positive values) or underestimation (negative values).

#### 2.2.2. Annual multivariate robustness analysis

To strengthen attribution and assess the robustness of the main findings, we complement the monthly counterfactual analysis with an annual multivariate robustness check, consistent with the standard empirical literature on cigarette demand. Given the addictive nature of tobacco consumption and the availability of key economic covariates at annual frequency, demand responses to prices and income are more appropriately modeled using annual data.

This analysis relies on a balanced annual provincial panel covering the period 2005–2025. The dependent variable is cigarette sales per capita, measured as the number of cigarette packs per adult per year and constructed from official legal sales and provincial population data. Explanatory variables include real cigarette prices (deflated using the general CPI, base year 2010), real GDP per capita, and the unemployment rate, all measured at the provincial level.

We estimate a panel interrupted time-series (ITS) model with province fixed effects, including a linear pre-intervention trend and a post-2011 level shift associated with the implementation of Law 42/2010. Standard errors are clustered at the provincial level to account for serial correlation. The general specification is Eq 6:

$$y_{p,t}=\alpha_{p}+\beta_{1}t+\beta_{2}Post2011_{t}+{\text{X}}_{p,t}\gamma +\varepsilon_{p,t},$$ (6)

where *y_p,t_* denotes the logarithm of cigarette sales per capita, *Post*2011_*t*_ is a dummy equal to one from 2011 onward, and *X_p,t_* is a vector of economic controls. Controls are introduced sequentially—first prices, then GDP per capita, and finally unemployment—to assess the stability of the estimated post-intervention effect. This annual analysis is explicitly conceived as a robustness and attribution check and does not replace the main high-frequency counterfactual framework.

## 3. Results

This section presents the main empirical results derived from the time-series modeling and forecasting analysis. The discussion follows a sequential logic: first, model validation and fit diagnostics; second, the counterfactual analysis for 2011–2013; third, the evaluation of cumulative impacts; and finally, the prospective scenario extending from 2025 onward.

### 3.1. Model performance and goodness of fit

Before estimating the counterfactual effects, the six forecasting models were compared in terms of predictive accuracy across all provinces.

**Figure 1.**
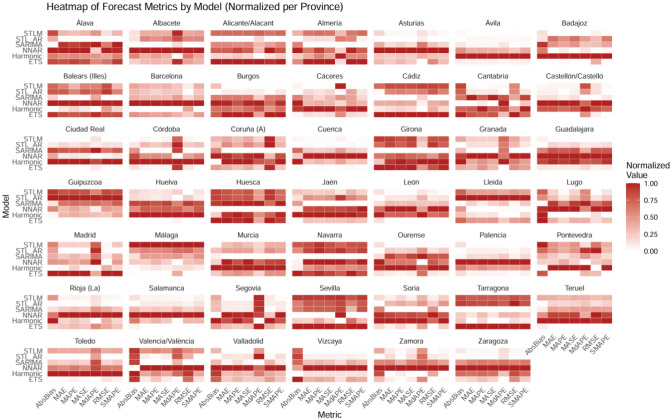
Normalized forecast error metrics by model and province.

Figure 1 displays a heatmap summarizing the forecast accuracy of the six time-series models (ETS, Harmonic, NNAR, SARIMA, STL_AR, and STLM) across all Spanish provinces. The figure reports normalized values for seven standard error and bias measures—AbsBias, MAE, MAPE, MASE, MdAPE, RMSE, and SMAPE—so that darker shades indicate higher (worse) normalized values, and lighter shades indicate better performance.

Overall, the visualization reveals a clear pattern of heterogeneity in model performance both across provinces and among forecasting approaches. The STLM and STL_AR models generally exhibit lighter tones across most metrics and regions, indicating superior fit and lower forecast errors. These models appear particularly robust in capturing complex seasonal and trend dynamics, benefiting from the local decomposition framework embedded in the STL family of models.

In contrast, the Harmonic and NNAR models display consistently darker cells, suggesting higher error magnitudes and less stable out-of-sample behavior. The ETS and SARIMA approaches occupy an intermediate position, offering relatively balanced accuracy but less adaptability to local nonlinearities.

At the national level, the predominance of lighter shading in the STLM and STL_AR rows supports their selection for the counterfactual and prospective analyses. Their superior goodness of fit indicates that they capture structural breaks and seasonality shifts more effectively, providing a reliable baseline for estimating the causal impact of the 2010 Anti-Smoking Law.

### 3.2. Counterfactual analysis (2011–2013)

Once the models were validated, counterfactual cigarette sales were estimated for each province over 2011–2013, representing the scenario in which the 2010 Anti-Smoking Law had not been implemented. As is standard in interrupted time-series designs, the estimated effects should be interpreted as policy-associated structural breaks rather than as fully isolated causal effects, particularly in the presence of concurrent economic and regulatory changes.

**Figure 2.**
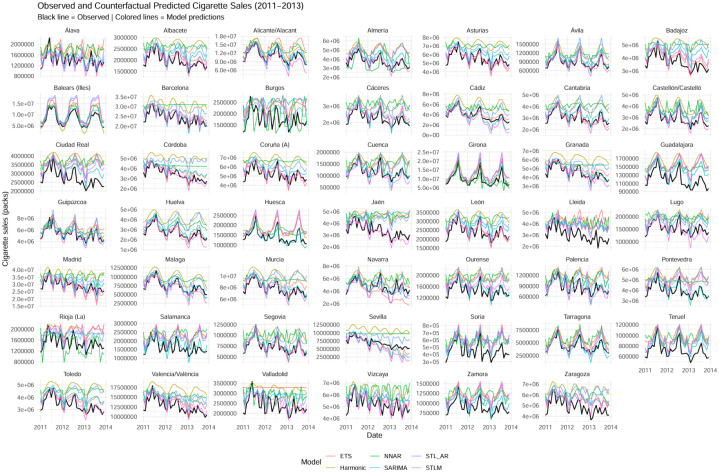
Observed and model-based counterfactual cigarette sales by province (2011–2013).

Figure 2 displays the observed and model-based counterfactual series of monthly cigarette sales for each province. The black lines represent observed sales, while the colored lines correspond to the six forecasting models used to construct the counterfactual scenarios. Each small multiple panel allows comparison of model performance and of the divergence between predicted (no-law scenario) and actual post-law sales.

Across nearly all provinces, the observed series lie below the counterfactual predictions throughout most of the post-law period, indicating that cigarette sales were consistently lower than would have been expected in the absence of regulation. This pattern provides strong visual evidence of the law's effectiveness in reducing tobacco consumption.

From a methodological perspective, the figure illustrates the coherence of the counterfactual estimates across models. Despite differences in functional form, the majority of models capture similar trajectories and turning points, with the STL_AR and STLM models producing smoother and more adaptive fits that align well with the observed data. This coherence reinforces the robustness of the modeling framework and the reliability of the estimated causal effect.

### 3.3. Evolution of the estimated effect (2011–2013)

The annual aggregation of monthly effects allows for an assessment of how the impact of the law evolved over time.

**Figure 3.**
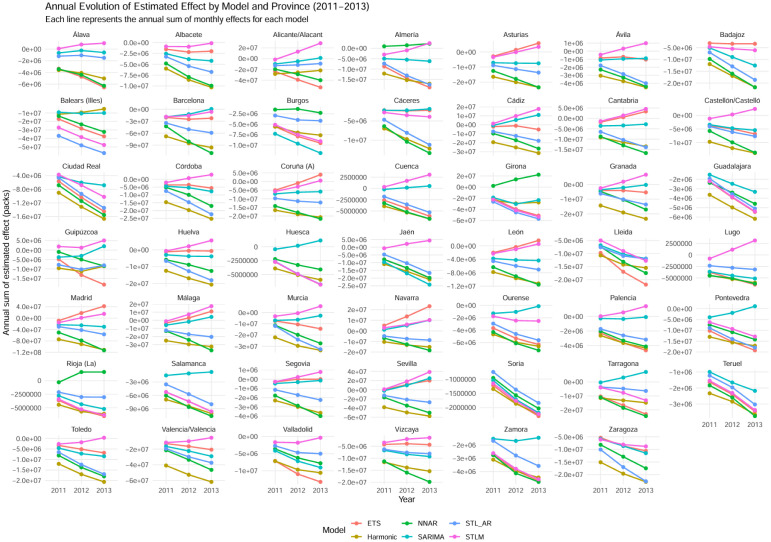
Declining annual effects of the 2010 Anti-Smoking Law by model and province (2011–2013).

Figure 3 shows the annual evolution of the estimated total effect derived from the monthly counterfactual models for 2011–2013. Each colored line corresponds to one forecasting approach, and each panel represents the aggregated annual trend. Across virtually all provinces and models, the lines display a consistent downward slope from 2011 to 2013, indicating that the estimated cumulative effect became increasingly negative over time.

This pattern suggests that the reduction in cigarette sales associated with the 2010 Anti-Smoking Law was not limited to an immediate response but persisted and deepened in subsequent years, reflecting structural adjustments in consumer behavior and the tobacco market.

Although some variation in slope magnitude appears across models, the overall direction of change is uniform. Models based on trend and seasonal decomposition (STL_AR and STLM) capture these persistent dynamics most effectively, while traditional specifications (ETS, SARIMA, Harmonic) produce slightly noisier but still consistently negative trajectories.

Overall, the figure confirms the sustained national impact of the law, with all models converging toward increasingly negative annual effects between 2011 and 2013.

### 3.4. Total cumulative impact (2011–2013)

The total effect of the 2010 Anti-Smoking Law was then computed by aggregating monthly impacts across provinces and models. Provincial effects are estimated in absolute terms, reflecting deviations in legal cigarette sales relative to the counterfactual scenario. National impacts are obtained by summing provincial effects across all provinces, ensuring that national estimates correspond to total volumes of legal cigarette sales.

Table 3 presents the total estimated impact of the law on cigarette sales, aggregated for 2011–2013. Negative values represent cumulative reductions relative to the counterfactual scenario without the law. Positive provincial values represent deviations relative to the counterfactual scenario rather than absolute post-policy increases in consumption. Such cases reflect expected heterogeneity and model sensitivity in provinces affected by tourism, border dynamics, or illicit market activity, and do not alter the aggregate national conclusions.

At the national level, the results indicate a pronounced and sustained decline in cigarette sales, regardless of the modeling approach. The total national effect ranges from −374 million packs (STLM model) to more than −2.45 billion packs (Harmonic model). Even the most conservative models, such as SARIMA (−695 million) and STL_AR (−1.69 billion), point to a substantial contraction in cigarette sales.

Although the STLM and STL_AR models display similarly strong in-sample goodness of fit, they produce different estimates of the cumulative national impact. This divergence reflects differences in how long-run counterfactual dynamics are extrapolated rather than disagreement about the existence or timing of the post-2011 structural break.

In the STL_AR specification, the remainder component is modeled using an autoregressive process, allowing pre-intervention persistence to propagate forward in the counterfactual scenario. This leads to a stronger continuation of pre-2011 dynamics and, consequently, a larger cumulative gap between observed and counterfactual sales. By contrast, the STLM model combines STL decomposition with exponential smoothing, which imposes greater trend regularization and adaptive updating, yielding a more conservative counterfactual trajectory and smaller cumulative impact estimates.

Taken together, these results highlight that differences across models primarily reflect alternative assumptions about long-run counterfactual behavior, while all specifications consistently identify a substantial and persistent decline in cigarette sales following the implementation of Law 42/2010.

These results imply that Law 42/2010 reduced between roughly 15% and 30% of total cigarette sales in Spain. The convergence of results across six diverse econometric frameworks provides strong evidence of a structural and persistent policy impact.

**Table 3.** Cumulative estimated impact of the 2010 Anti-Smoking Law by model and province (2011–2013).

Province	ETS	Harmonic	NNAR	SARIMA	STL_AR	STLM
Álava	−14,637,469.09	−12,723,272.15	−14,133,470.86	−1,494,409.197	−3,785,700.262	1,863,280.114
Albacete	−5,757,162.801	−24,888,038.42	−22,693,383.36	−10,633,116.87	−15,468,759.19	−2,027,803.56
Alicante/Alacant	−116,062,645	−75,666,288.84	−88,437,952.7	−13,600,254.89	−33,818,545.03	39,897,910.78
Almería	−42,111,003.33	−44,668,886.36	4,742,533.141	−16,669,828.88	−38,050,963.42	−1,429,088.853
Asturias	4,186,064.283	−59,848,925.13	−53,815,655.45	−22,189,281.63	−33,970,767.72	−329,974.1209
Ávila	−2,601,198.501	−11,281,018.78	−9,956,015.82	−2,946,191.463	−8,622,277.703	860,572.7447
Badajoz	−9,750,939.14	−50,654,073.88	−47,618,058.49	−26,584,919.83	−38,542,470.23	−16,137,668.29
Balears (Illes)	−82,954,105.95	−26,323,203.63	−69,345,641.99	−29,890,719.15	−142,080,849.4	−113,247,302
Barcelona	−68,484,309.3	−249,147,981.5	−233,470,176.2	−31,162,685.69	−144,072,509.5	−41,615,051.12
Burgos	−23,041,523.37	−20,251,818.49	−5,122,327.333	−28,891,591.66	−11,072,297.27	−21,710,144.74
Cáceres	−6,739,947.112	−28,917,648.5	−30,167,617.5	−6,316,299.759	−23,967,200.81	−10,147,836.98
Cádiz	−8,248,061.046	−75,983,089.87	−53,264,406.66	15,754,831.27	−37,592,736.69	28,785,257.51
Cantabria	2,146,248.297	−34,201,885.93	−38,493,068.49	−9,838,726.333	−30,765,659.4	4,648,765.95
Castellón/Castelló	−15,633,252.84	−35,664,588.97	−29,376,987.73	−13,619,041.01	−17,877,268.37	1,549,823.381
Ciudad Real	−30,532,943.61	−38,817,329.56	−33,811,570.85	−17,235,791.67	−27,449,736.84	−20,780,061.13
Córdoba	−12,804,328.05	−59,665,488.84	−32,167,724.22	−17,523,831.36	−44,523,624.9	1,635,518.662
Coruña (A)	−1,964,419.257	−56,258,856.18	−53,759,851.62	−19,188,521.77	−33,270,477.08	−8,211,055.456
Cuenca	−13,178,423.57	−16,044,246.31	−15,261,429.56	490,175.1901	−10,691,035.82	5,050,043.374
Girona	−111,225,659.3	−80,283,959.29	38,436,769.12	−72,531,530.29	−128,553,561.9	−117,243,611
Granada	−13,583,679.33	−56,799,315.68	−32,245,166.63	−6,065,550.786	−30,019,526.25	5,954,120.585
Guadalajara	−11,643,506.12	−14,803,279.08	−10,362,875.89	−7,317,682.39	−10,597,869.42	−11,465,036.65
Guipúzcoa	−36,420,395.48	−29,499,764.69	−14,444,146.18	−4,837,611.827	−26,193,876.8	8,246,169.903
Huelva	−2,440,784.229	−49,916,071.84	−27,271,181.47	−10,734,963.13	−37,274,739.15	6,952,798.933
Huesca	−14,465,508.79	−14,934,132.02	−9,591,267.661	855,869.7099	−14,288,465.53	−14,414,324.15
Jaén	−41,220,451.22	−47,354,985.3	−41,162,111.22	−50,735,016.09	−31,728,040.62	5,931,000.216
León	−787,855.5604	−28,666,486.39	−26,966,303.17	−12,354,444.38	−17,594,456.63	−2,752,723.16
Lleida	−48,540,391.92	−40,338,317.98	−37,893,321.79	−28,960,502.25	−30,429,456.8	−26,660,328.07
Lugo	−15,356,100.22	−15,745,577.36	−15,811,528.46	−13,271,275.67	−8,286,602.868	3,492,701.221
Madrid	50,499,602.61	−277,641,973.3	−242,353,607.5	−78,997,619.46	−129,205,904.4	217,275.6589
Málaga	10,554,751.83	−86,754,185.14	−74,104,231.96	−3,385,661.982	−49,920,667.66	23,440,980.45
Murcia	−32,564,539.78	−85,340,981.56	−58,569,112.69	−16,200,955.2	−68,550,181.88	1,736,648.689
Navarra	42,023,541.3	−38,841,309.86	−37,934,436.63	16,445,852.79	−21,042,500.57	19,144,978.83
Ourense	−15,309,368.15	−17,252,260.25	−17,690,915.88	−2,432,835.852	−13,173,673.78	−6,702,273.356
Palencia	−10,578,538.38	−10,630,228.01	−9,503,515.131	−719,420.7413	−7,515,413.658	1,966,513.18
Pontevedra	−44,721,902.19	−45,730,948.48	−32,551,988.32	−4,842,004.84	−40,930,450.17	−28,498,209.76
Rioja (La)	−15,656,685.74	−16,221,248.54	2,915,285.346	−12,329,137.56	−8,159,284.027	−15,022,966.79
Salamanca	−22,334,955.83	−25,530,998.66	−25,394,082.82	−3,334,439.819	−17,300,553.12	−22,325,331.79
Segovia	−327,924.8466	−8,972,679.282	−8,667,170.956	−949,388.4571	−5,130,935.848	711,923.821
Sevilla	31,113,794.84	−145,320,195.1	−101,617,719.2	32,236,968.56	−61,461,992.48	57,484,852.13
Soria	−5,349,251.521	−5,445,498.402	−4,557,808.552	−4,939,365.681	−3,958,719.142	−5,137,290.118
Tarragona	−50,157,299.64	−39,627,416.43	−54,071,392.37	9,991,446.566	−14,858,827.19	−24,867,005.47
Teruel	−7,483,861.771	−8,824,510.239	−8,178,690.509	−4,841,636.903	−6,216,345.08	−7,222,377.859
Toledo	−15,295,479.4	−49,776,172.28	−39,857,707.85	−20,300,595.67	−35,978,944.82	−4,140,750.961
Valencia/València	−50,041,281.17	−155,418,151	−101,538,027.4	−67,996,041.53	−86,625,736.35	−26,912,655.58
Valladolid	−31,271,994.8	−27,387,420.04	−17,618,818.96	−20,338,183.85	−12,392,361.67	−3,825,470.61
Vizcaya	−12,431,789.38	−40,842,169.08	−47,057,658.54	−24,265,669.72	−21,905,726.38	−6,829,683.99
Zamora	−11,370,643.38	−11,533,814.65	−11,672,383.38	−4,642,013.894	−8,034,774.054	−10,997,609.17
Zaragoza	−24,522,871.29	−57,610,268.35	−38,527,304.87	−25,938,757.95	−49,789,368.06	−22,953,226.38
Tootal (Spain)	−959,080,448.2	−2,454,050,960	−1,932,015,227	−695,272,373	−1,692,741,836	−374,035,724.9

Although magnitudes vary regionally, the pattern remains homogeneous across the territory, confirming that the regulation produced a broad, sustained contraction in the Spanish tobacco market.

### 3.5. Prospective scenario (2025–2027)

Finally, the validated models were used to project cigarette sales from August 2025 through December 2027, assuming no additional policy changes other than the continuation or reinforcement of existing restrictions. The projections for the 2025–2027 period correspond to prospective simulations generated by the validated forecasting models and do not represent observed data.

**Figure 4.**
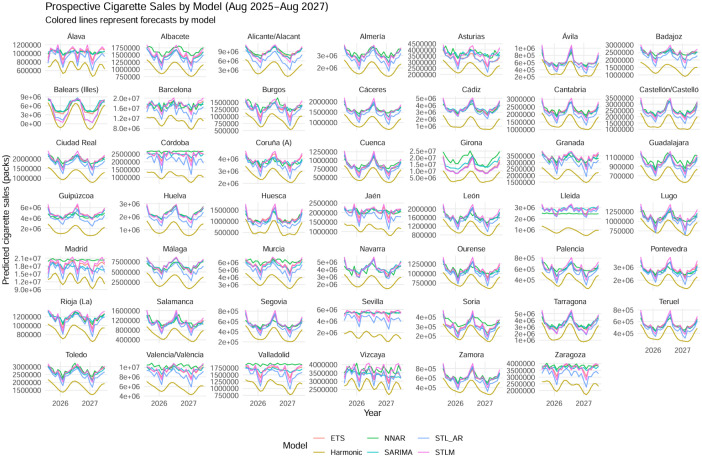
Projected cigarette sales under alternative forecasting models (2025–2027).

Figure 4 presents model-based projections of monthly cigarette sales for the 2025–2027 period. Each colored line corresponds to one of the six forecasting models, and each panel represents the forecast trajectory at the provincial level.

Across all models, the projected series display consistent cyclical patterns and moderate downward trends, suggesting stabilization of cigarette sales at levels below the pre-2025 baseline. No model anticipates a recovery in tobacco consumption during the projection horizon, reinforcing the interpretation that the effects of the 2010 Anti-Smoking Law persist in the medium term.

Model dispersion is limited, with the STL-based and SARIMA models producing the smoothest and most stable projections, while Harmonic and NNAR generate slightly more volatile series. Nevertheless, all forecasts align in direction, indicating the continued persistence of reduced cigarette sales.

Overall, the figure depicts a scenario of ongoing stabilization or gradual decline in tobacco consumption through 2027. The convergence of forecasts across methodologies suggests that the sustained enforcement of anti-smoking measures would maintain, rather than reverse, the downward trajectory established after 2010.

### 3.6. Annual multivariate robustness check

Table 4 reports the results of the annual panel ITS models. Across all specifications, the coefficient associated with the post-2011 indicator remains negative and highly statistically significant, indicating a robust structural break coinciding with the implementation of the comprehensive smoking ban.

**Table 4.** Annual panel ITS robustness check (2005–2025).

Model	Controls included	Post-2011 coefficient (β₂)	Approx. % change	p-value
(1) Baseline ITS	None	−0.343***	−29.0%	1e−100
(2) + Price	Real price	−0.152***	−14.1%	3.17e−10
(3) Full model	Price + GDPpc + Unemployment	−0.115***	−10.8%	1.10e−12

Notes: Percentage changes are computed as $100\times (e^{\beta_{2}}-1)$. *** significance at 1% level. ** significance at 5% level. * significance at 10% level.

The estimated post-2011 decline ranges from approximately 29% in the baseline specification to around 11% in the fully controlled model, indicating that while part of the observed reduction is associated with pricing and macroeconomic conditions, a substantial and statistically significant decline persists after accounting for these factors.

### 3.7. Provincial heterogeneity by structural typologies

To explore potential heterogeneity in the estimated effects, provinces were classified according to the structural typologies proposed by Cadahia et al. [36]. Following this classification, touristic provinces include Alicante, Almería, Balears, Málaga, and Tarragona; border provinces comprise Girona, Guipúzcoa, Huesca, Lleida, Navarra, and Teruel; and provinces with higher exposure to illicit trade and smuggling include Cádiz, Córdoba, and Sevilla. All remaining provinces are grouped as “other provinces”. These classifications are fixed over time and defined ex ante.

The heterogeneity analysis is based on the STL_AR specification, which provides stable and conservative provincial estimates and is well-suited for cross-regional comparisons. The estimated post-2011 decline in cigarette sales is observed across all provincial typologies, although the magnitude of the reduction differs across groups (Table 5). Touristic provinces exhibit the largest average decline, followed by provinces with higher exposure to illicit trade and border provinces, while the remaining provinces also show a substantial negative effect. These differences indicate heterogeneity in intensity rather than in the direction of the policy impact and do not alter the main conclusions of the study.

**Table 5.** Average post-2011 impact by provincial typology (STL_AR).

Provincial typology	Number of provinces	Average impact	Median impact
Touristic	5	−55.7	−38.1
Border	6	−37.8	−23.6
Illicit trade exposure	3	−47.9	−44.5
Other provinces	34	−30.7	−19.9

Notes: Impacts correspond to cumulative post-2011 effects estimated using the STL_AR model. All values are negative, indicating a decline in legal cigarette sales across all provincial typologies.

## 4. Discussion

This study provides empirical evidence of the causal and predictive impact of Spain's 2010 Anti-Smoking Law on cigarette sales, using a hybrid time-series and machine learning framework applied to monthly provincial data spanning 2005–2027. By combining counterfactual inference with prospective simulation, the analysis captures both the historical and forward-looking dimensions of policy effectiveness. The findings confirm that the law generated an immediate, substantial, and sustained reduction in cigarette sales across Spain, with no signs of market recovery in the medium term.

The consistency of results across models—spanning classical econometric (SARIMA, ETS), nonlinear (NNAR), and hybrid decomposition approaches (STL_AR, STLM)—strengthens the robustness of the conclusions. The fact that each model family, despite their differing assumptions, yields comparable estimates in both magnitude and direction implies that the observed decline is not an artifact of model specification. Rather, it reflects a genuine structural shift in consumer behavior and market dynamics. This convergence supports the broader interpretation that comprehensive anti-smoking laws, when fully enforced, produce enduring effects that extend beyond short-term compliance or temporary deterrence.

From a public health perspective, these findings align with international evidence demonstrating that comprehensive tobacco control legislation leads to significant and persistent declines in cigarette consumption [3],[9]. The estimated national reduction—between 0.37 and 2.45 billion cigarette packs over the first three post-law years—represents one of the most substantial market contractions documented in the European context. This confirms that Law 42/2010, by prohibiting indoor smoking and tightening advertising restrictions, achieved a level of effectiveness comparable to similar comprehensive bans implemented in Northern and Western Europe.

Methodologically, the integration of hybrid time-series and machine learning techniques advances the field of policy evaluation in public health. The results highlight that model ensembles leveraging both interpretability (SARIMA, ETS) and flexibility (NNAR, STLM) outperform single-model approaches in identifying structural breaks and seasonal changes associated with behavioral regulation. This hybrid framework allows researchers to quantify not only realized impacts but also potential future trajectories under alternative policy conditions. The ability to extend the counterfactual structure into a prospective horizon (2025–2027) transforms conventional retrospective evaluation into predictive policy analytics—an emerging paradigm for anticipatory governance in public health [17],[25].

The prospective projections indicate that cigarette sales are expected to stabilize or decline slightly through 2027, even without new policy interventions. This suggests that the behavioral and cultural shifts initiated by the 2010 law have become embedded in the social fabric. However, the projections also imply that without renewed regulatory measures or intensified enforcement, the pace of decline may plateau, highlighting the need for sustained public health engagement. Complementary interventions—such as plain packaging, expanded smoke-free zones, and digital advertising bans—could reinforce the downward trend and prevent normalization of tobacco use among younger cohorts.

Despite the strength of these findings, several limitations merit consideration. First, the models rely on official sales data and do not capture potential substitution effects toward illicit or alternative products (e.g., vaping or roll-your-own tobacco). Second, while model diversity mitigates overfitting and specification bias, the absence of external covariates such as taxation, income, or enforcement intensity may limit causal precision. Future research could integrate multivariate time-series or Bayesian structural models to estimate the interactive effects of policy, price, and socioeconomic factors. Third, although the models achieve high predictive accuracy, the translation from sales data to consumption behavior remains inferential; household-level surveys would provide complementary validation.

Further consideration concerns potential substitution toward alternative nicotine products, particularly e-cigarettes. While this mechanism has been shown to play a role in other contexts [37], its relevance in Spain during the period analyzed appears limited. First, there are no official administrative data on e-cigarette sales in Spain, as these products are not derived from the tobacco leaf and therefore fall outside the regulatory and statistical scope of the Trade of Tobacco Commission. Second, available evidence indicates that the diffusion of alternative nicotine products in Spain was minimal during the years immediately following the implementation of Law 42/2010. Martín-Álvarez et al. [38] documented that heated tobacco products and related alternatives accounted for less than 2% of the Spanish market as late as 2016, implying a negligible presence around 2010–2011.

Consequently, while some degree of substitution toward alternative products cannot be ruled out, it is unlikely to account for the magnitude and timing of the sharp decline in legal cigarette sales observed after 2011. The results are therefore best interpreted as documenting a persistent contraction of the legal cigarette market associated with the comprehensive smoking ban, rather than as a direct measure of smoking cessation or health outcomes.

The annual multivariate robustness analysis reinforces the interpretation derived from the main monthly counterfactual framework. Even after controlling for standard demand-side fundamentals such as prices, income, and labor market conditions, a significant structural break in cigarette sales per capita persists following the implementation of Law 42/2010. The convergence of results across frequencies and modeling strategies indicates that the observed contraction of the legal cigarette market cannot be attributed solely to macroeconomic trends or pricing dynamics. Rather, the findings are consistent with a substantial and sustained policy-associated decline in cigarette sales, strengthening the overall credibility of the study's conclusions.

The sales-based results are consistent with independent epidemiological evidence from national health surveys. Using microdata from the Spanish National Health Survey and the European Health Interview Survey, Martín-Álvarez et al. [39] documented a sustained decline in daily smoking prevalence in Spain following the implementation of comprehensive smoke-free legislation, with prevalence falling by more than four percentage points between 2006 and 2017. Although prevalence and sales capture different dimensions of tobacco use, the parallel downward trends observed across data sources provide external validation for the direction and persistence of the estimated effects.

The interpretation of the estimated effects warrants careful consideration of potential threats to attribution. The period surrounding the implementation of Law 42/2010 coincided with several relevant developments, including changes in tobacco taxation and prices, the economic crisis, demographic and tourism dynamics, substitution toward alternative nicotine products, illicit market activity, and the presence of overlapping regulatory milestones such as Law 28/2005.

While these factors may have contributed to the long-run evolution of cigarette sales, several elements support a substantive role for the comprehensive smoking ban. First, the estimated decline is temporally aligned with the implementation of Law 42/2010 and displays an immediate and persistent pattern characteristic of structural policy interventions. Second, results are consistent across a wide range of econometric and machine-learning specifications, reducing concerns about model-driven artifacts. Third, an annual multivariate robustness analysis incorporating real prices, income, and labor market conditions confirms that a statistically significant post-2011 break remains after conditioning on standard demand-side fundamentals.

Taken together, these findings suggest that the observed contraction of the legal cigarette market cannot be explained solely by macroeconomic trends or pricing dynamics. Rather, the evidence supports a cautious interpretation in which Law 42/2010 played a central role within a broader set of contemporaneous influences. Accordingly, the results should be interpreted as bounding the policy-associated impact of the smoking ban rather than attributing the entire observed decline to a single mechanism.

Overall, this study contributes to the growing literature on computational public health by demonstrating that hybrid counterfactual modeling can bridge epidemiological evaluation and policy simulation. The framework applied here is generalizable to other public health domains—such as alcohol regulation, sugar-sweetened beverage taxes, or environmental restrictions—where policymakers require ex-ante evaluation of alternative scenarios.

## 5. Conclusions

This study provides robust evidence that the implementation of Spain's comprehensive smoking ban was associated with an immediate and persistent reduction in legal cigarette sales, as measured using high-frequency provincial data. By combining counterfactual time-series methods and prospective forecasting, the analysis documents a substantial contraction of the legal tobacco market following the introduction of Law 42/2010.

Importantly, the results should be interpreted in light of the outcome directly observed in the data. Legal cigarette sales capture market-level and behavioral responses to regulation and serve as a widely used proxy for tobacco consumption, but they do not allow for a direct quantification of health outcomes or broader macroeconomic effects. Accordingly, the findings are best understood as evidence of sustained changes in legal sales dynamics rather than as direct estimates of health impacts.

Nevertheless, the persistence and magnitude of the observed decline are consistent with a broader body of evidence documenting the effectiveness of comprehensive smoke-free policies. Future research combining sales data with epidemiological or health outcome measures could further explore the downstream implications of these market-level effects.

The evidence presented confirms that Spain's 2010 Anti-Smoking Law produced a strong, lasting, and nationwide reduction in cigarette sales. The observed and counterfactual analyses consistently show that the legislation's impact extended well beyond its initial enforcement period, shaping long-term market and behavioral dynamics. All model families—statistical, hybrid, and machine learning—converge in demonstrating a persistent decline in cigarette consumption between 2011 and 2013, followed by a stable or slightly decreasing trend projected through 2027.

At the national level, the estimated contraction of between 0.37 and 2.45 billion cigarette packs underscores the law's effectiveness as a major public health intervention. The absence of a rebound in sales in the prospective horizon further supports the hypothesis that the reform has induced structural change rather than temporary behavioral adjustment.

From a methodological standpoint, the study demonstrates that combining SARIMA, STL-based, and neural network models provides a reliable and flexible framework for assessing public health interventions. Beyond tobacco control, this integrative approach exemplifies how predictive and counterfactual analytics can inform the design, timing, and evaluation of future policies.

In conclusion, Spain's experience with comprehensive anti-smoking legislation illustrates the transformative potential of evidence-based policy. When enforced consistently and supported by ongoing public health communication, such measures not only achieve immediate reductions in harmful behaviors but also sustain long-term cultural and economic benefits. The results highlight the importance of maintaining and expanding these efforts to consolidate the gains achieved and to guide future interventions aimed at achieving a smoke-free generation.

## Use of AI tools declaration

The authors declare they have not used Artificial Intelligence (AI) tools in the creation of this article.
